# A Review of the Evidence of Harm from Self-Tests

**DOI:** 10.1007/s10461-014-0831-y

**Published:** 2014-07-03

**Authors:** Annette N. Brown, Eric W. Djimeu, Drew B. Cameron

**Affiliations:** The International Initiative for Impact Evaluation, 1625 Massachusetts Ave., NW Suite 450, Washington, DC 20036 USA

**Keywords:** HIV self-testing, Harm, Self examinations, Self tests

## Abstract

Although HIV self-testing may overcome some barriers to HIV testing, various stakeholders have expressed concerns that HIV self-testing may lead to unintended harm, including psychological, social and medical harm. Recognizing that similar concerns were raised in the past for some other self-tests, we conduct a review of the literature on a set of self-tests that share some characteristics with HIV self-tests to determine whether there is any evidence of harm. We find that although the potential for harm is discussed in the literature on self-tests, there is very little evidence that such harm occurs.

## Introduction

HIV testing is the gateway to treatment and care for those infected with HIV and key to many prevention interventions such as behavior change communications to reduce risky behavior, treatment as prevention (providing treatment to prevent transmission of the virus to an HIV negative partner) and prevention of mother to child transmission (PMTCT). Although different approaches to HIV testing, including voluntary counseling and testing, provider-initiated counseling and testing, mobile testing and home-based testing, have been shown to increase the prevalence of HIV testing [1–3], HIV self-testing may be able to reach individuals who have never tested as well as offer a convenient alternative for repeat testing.

In spite of considerable optimism for HIV self-testing voiced by a variety of stakeholders, including donors and program implementers, support is not universal. The World Health Organization (WHO) published the “First international symposium on HIVST: legal, ethical, social, gender and public health implications of HIVST scale-up” meeting report which states, “HIVST remains a concern for many policymakers and implementers due to the associated ethical, legal, and social issues” [4]. Among these issues are concerns about potential unintended harm, including psychological harm when testing and counseling are decoupled, social harm from the potential unethical use of HIV self-test kits (coercion/undue influence) or from a nonreactive (negative) HIV self-test resulting in justification for unprotected sex, and medical harm from greater potential for inaccurate results [5–8].

Recognizing that some of these same concerns have been raised previously about other self-tests, we conducted a review of the literature to look for evidence of actual harm from other selected self-tests as well as from HIV self-tests.

## Methods

We conducted a search of the following eight major academic databases: Embase (Ovid), Medline (Ovid), PsycINFO (Ovid), CAB Abstracts (Ovid), Africa Wide Info (Ebsco), SocIndex (Ebsco), Academic Search Complete (Ebsco) and the Cochrane Library. We used the following terms: self test(ing/s), self diagnostic(s), home based (test(s/ing), screen(ing), diagnostic(s), evaluation(s) and examination(s)), self examination(s), self screen(ing) and (self/home based) rapid diagnostic test(s). We searched using the title, abstract and keyword fields and limited results to English language results. Search results included both academic and non-academic (trade journals, magazines, newspapers, etc.) articles. There were no geographic limitations to the search. Articles published prior to 1990 were not reviewed in order to prioritize more recent evidence. Articles were included up to 14 August 2013 for all tests except pregnancy tests, for which the search was extended up to 27 January 2014 as additional terms were added following the initial screen.

**Fig. 1:**
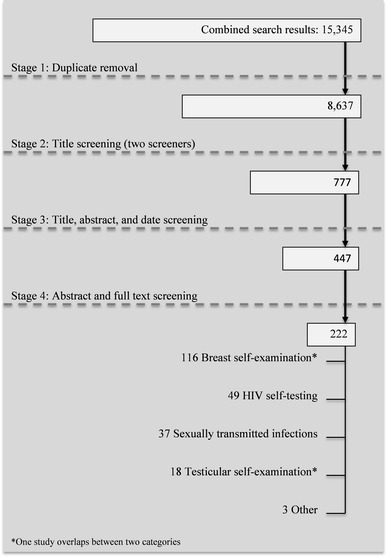
Screening flow chart of search results from 8 to 14 August 2013

**Fig. 2:**
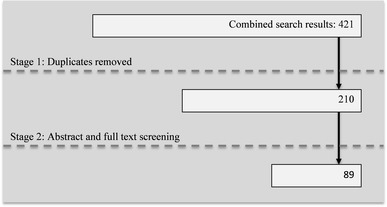
Screening flow chart of search results from 23 to 27 January 2014

Self-tests for HIV, sexually transmitted infections (STIs), testicular self-examination (TSE), breast self-examination (BSE) and home pregnancy tests were included in this screening protocol. This list was developed to (1) focus on self-tests whereby the user learns the result at home and is not directly linked to face-to-face counseling and (2) include information on self-testing for diseases that might be fatal or carry negative stigma or serious anxiety. Therefore, monitoring tests, such as those for international normalized ratio (INR) testing (anti-coagulate) or blood sugar testing for type I or II diabetes, were excluded since these tests are part of self-management programs for patients who already know they have the disease. Evidence of psychological, social and medical harm for the included self-tests was sought in this review (Figs. 1, 2).

## Results

Table 1 presents the number of hits from the search for each database. From the two searches a total of 8,847 titles were screened and 311 articles from the two searches selected for full-text screening. Forty-nine of these studies were on HIV self-testing. Of those, a little more than one quarter mention anxiety, fear or worry in relation to self-testing, but this was in relation to a potential reduction of such emotions through the use of self-testing by eliminating the waiting time associated with other methods of HIV testing. Around a quarter of articles mention the potential concern of suicide in relation to HIV self-testing, but no evidence of suicide after an HIV self-test was reported. Two studies explore the possibility of social harm [9, 10] but the authors conclude they are not aware of any evidence of such harm having occurred. Less than one third of the articles mentioned the possibility of false negative results from HIV self-tests. However, there was no evidence of actual harm occurring due to false negative results.

**Table 1:** Search results by database

Name of database	Search results for “self-testing” search 8–14 August 2013	Search results for “pregnancy self-testing” search 23–27 January 2014
Embase (Ovid)	3,950	174
Medline (Ovid)	3,397	108
PsycINFO (Ovid)	1,534	20
CAB Abstracts (Ovid)	245	5
Africa Wide Info (Ebsco)	535	7
SocIndex (Ebsco)	798	12
Academic Search Complete (Ebsco)	4,368	83
Cochrane Library	718	12
Sum	15,345	421
Total (after duplicates removed)	8,637	210

Source: Author constructed

Thirty-seven articles on self-tests for other STIs were screened, and again none provided evidence of harms occurring. Three articles list anxiety, fear or worry as barriers to individuals seeking testing, and two suggest a correlation between home-based chlamydia testing and engaging in sexually risky behavior, but no evidence supporting a causal relationship was provided.

One hundred and sixteen articles on BSE were reviewed. Roughly one third discuss anxiety, fear or worry surrounding breast cancer and BSE; many of these focus on anxiety related to risk factors for breast cancer such as a family history. While a few suggest that these emotions can be a barrier to conducting BSE [11, 12], others, including a meta-analysis covering 12 studies [13], show evidence that breast cancer worry increases screening behaviors. We found no evidence of anxiety arising solely from performing BSE. No study mentioned social harm. A few articles discuss the possibility for false positives and suggest that harm, such as increased costs to health care systems, could result [14–22]. However, these articles do not present evidence of harm actually occurring due to false positives.

Eighteen articles on TSE were reviewed. Roughly half suggest that the exam could cause anxiety, though no evidence of such harm occurring was provided. One article mentioned the possibility of increased costs due to a false positive, similar to the argument for BSE, but without evidence of such harm occurring.

Of 89 articles on home pregnancy tests reviewed, roughly one third mention the possibility of a false negative result in the context of test accuracy, but none presents evidence of harm occurring as a result of a false negative.

In terms of psychological harm, across the 311 articles screened in full text, the studies of BSE and TSE include the most discussion of potential anxiety or worry due to the exam. The survey results reported in several BSE studies suggest that the practice of BSE can cause anxiety (independent of the whether a lump is felt) and that worry or fear are barriers to conducting the exam in the first place. However, there is no evidence of serious psychological consequences from having conducted the exam [11–13, 23–28].

Social harm only appears as a potential concern for HIV self-testing and self-testing for other STIs. The articles on HIV self-testing that use the word “risk” generally refer to “at-risk” populations or “risk reduction”. None of the articles on self-testing for STIs discusses coercion or presents evidence of risk behavior as a result of self-testing. Additionally, there was no reported evidence of coercive use of home pregnancy tests.

The medical harm discussed across the full set of articles includes harm arising from false negative and from false positive self-test results. Discussion of false negative results appears most often in the studies on HIV self-testing. One reason false negative results are a concern for self-testing is that there is a longer period during which a new infection is not detected for the oral self-test than for conventional blood tests, so there is an increased likelihood of false negatives [29–34]. No studies provide evidence of treatment delay due to a false negative test result.

Concerns about false positive test results arise in some studies on BSE and TSE. Some studies, particularly for BSE, suggest that false positive results may lead to increased costs to health care systems through unnecessary medical testing [14, 26–28]. However, none of the reviewed articles presents evidence that BSE has indeed caused significant unnecessary testing and costs. There is also mention in some articles of psychological harm—unnecessary anxiety or worry—that may arise from false positive TSE and BSE exam results. But there is no evidence presented that such harm is highly prevalent or consequential. Regarding false negative results, there is no evidence presented to suggest that false negative results from self-exams are an important factor in treatment delay.

## Discussion

The main finding from review of over 300 articles is that there is little if any evidence of the three types of harm (psychological, medical or social) from the reviewed self-tests. While many articles use words such as anxiety, risk behavior or inaccuracy, these are usually used in the context of seeking self-testing and the varying sensitivity and specificity estimates for the different test types.

The objective of this exercise was to uncover any notable evidence of harm resulting from HIV self-tests or other self-tests that share similar characteristics in order to address current concerns regarding HIV self-testing. This review employed a broad search strategy and screened a large number of articles, and as a result, some evidence may have been missed.

Despite the limitations, this review found very little evidence of any harm occurring in the practice of self-testing. Based on these findings, we recommend that HIV self-testing not be restricted based on fears of harm, but rather that as self-testing is expanded, researchers and policy makers pay particular attention to monitoring and measuring for unintended harm.

## References

[1] Sabapathy K, Van den Bergh R, Fidler S, Hayes R, Ford N (2012). Uptake of home-based voluntary HIV testing in sub-Saharan Africa: a systematic review and meta-analysis. PLoS Med.

[2] Suthar AB, Ford N, Bachanas PJ (2013). Towards universal voluntary HIV testing and counselling: a systematic review and meta-analysis of community-based approaches. PLoS Med.

[3] Kennedy CE, Fonner VA, Sweat MD, Okero FA, Baggaley R, O’Reilly KR (2013). Provider-initiated HIV testing and counseling in low- and middle-income countries: a systematic review. AIDS Behav.

[4] Report on the first international symposium on self-testing for HIV: the legal, ethical, gender, human rights and public health implications of HIV self-testing scale up. Geneva, Switzerland: World Health Organization; Apr 2013. Available from: http://www.who.int/hiv/pub/vct/self_test/en/. Accessed on 2014 May 22.

[5] United States Food and Drug Administration (FDA), Department of Health and Human Services. 85th Meeting of Blood Products Advisory Committee. Gaithersburg, MD: CASET Associates, November 3, 2005. Available at: http://www.fda.gov/ohrms/dockets/ac/05/transcripts/2005-4190t1.htm. Accessed on 2014 May 22.

[6] Spielberg F, Levine RO, Weaver M. Home self-testing for HIV: direction for action research in developing countries. Seattle, WA: The Synergy Project (US); Jun 2003 10. Available from: http://pdf.usaid.gov/pdf_docs/PNACW413.pdf. Accessed on 2014 May 23.

[7] Wright AA, Katz IT (2006). Home testing for HIV. N Engl J Med.

[8] Spielberg F, Levine RO, Weaver M (2004). Self-testing for HIV: a new option for HIV prevention?. Lancet Infect Dis.

[9] PantPai N, Klein MB (2008). Are we ready for home-based, self-testing for HIV?. Futur HIV Ther.

[10] Campbell S, Klein R (2006). Home testing to detect human immunodeficiency virus: boon or bane?. J Clin Microbiol.

[11] Posluszny DM, McFeeley S, Hall L, Baum A (2004). Stress, breast cancer risk, and breast self-examination: chronic effects of risk and worry. J Appl Biobehav Res.

[12] Kearney AJ (2006). Increasing our understanding of breast self-examination: women talk about cancer, the health care system, and being women. Qual Health Res.

[13] Hay JL, McCaul KD, Magnan RE (2006). Does worry about breast cancer predict screening behaviors? a meta-analysis of the prospective evidence. Prev Med.

[14] Smith RA, Cokkinides V, Eyre HJ (2005). American Cancer Society guidelines for the early detection of cancer, 2005. CA Cancer J Clin.

[15] Beacham AO, Carpenter JS, Andrykowski MA (2004). Impact of benign breast biopsy upon breast self-examination. Prev Med.

[16] Danish SJ, Chopin SM, Conley K (2008). Rethinking breast self-examinations: are we asking the right questions?. Breast Cancer (Auckl).

[17] Brain K, Norman P, Gray J, Mansel R (1999). Anxiety and adherence to breast self-examination in women with a family history of breast cancer. Psychosom Med.

[18] Breast self-exams: Some now say they are of no value. Mod Med. 1995;63(8):8. Available from: http://connection.ebscohost.com/c/articles/9601153644/breast-self-exams-some-now-say-they-are-no-value. Accessed on 2014 May 22.

[19] Absetz P, Aro AR, Sutton SR (2003). Experience with breast cancer, pre-screening perceived susceptibility and the psychological impact of screening. Psychooncology..

[20] Moulds A (2000). The dangers of self-examination. Practitioner.

[21] Tarrant M (2006). Why are we still promoting breast self-examination?. Int J Nurs Stud.

[22] Thomas DB, Gao DL, Ray RM (2002). Randomized trial of breast self-examination in Shanghai: final results. J Natl Cancer Inst.

[23] Katapodi MC, Lee KA, Facione NC, Dodd MJ (2004). Predictors of perceived breast cancer risk and the relation between perceived risk and breast cancer screening: a meta-analytic review. Prev Med.

[24] Moore P (1999). Excessive breast self-examination may promote anxiety. Lancet.

[25] Ceber E, Soyer MT, Ciceklioglu M, Cimat S (2006). Breast cancer risk assessment and risk perception on nurses and midwives in Bornova Health District in Turkey. Cancer Nurs.

[26] Spiegel TN, Hill KA, Warner E (2009). The attitudes of women with BRCA1 and BRCA2 mutations toward clinical breast examinations and breast self-examinations. J Womens Health (Larchmt).

[27] Hussein DM, Alorf SH, Al-Sogaih YS (2013). Breast cancer awareness and breast self-examination in Northern Saudi Arabia: a preliminary survey. Saudi Med J.

[28] Nahcivan NO, Secginli S (2007). Health beliefs related to breast self-examination in a sample of Turkish women. Oncol Nurs Forum.

[29] Bilardi JE, Walker S, Read T (2013). Gay and bisexual men’s views on rapid self-testing for HIV. AIDS Behav.

[30] PantPai N, Sharma J, Shivkumar S (2013). Supervised and unsupervised self-testing for HIV in high- and low-risk populations: a systematic review. PLoS Med.

[31] Wilson DP, Fairley CK, Sankar D (2011). Replacement of conventional HIV testing with rapid testing: mathematical modeling to predict the impact on further HIV transmission between men. Sex Transm Infect.

[32] Owen SM, Yang C, Spira T (2008). Alternative algorithms for human immunodeficiency virus infection diagnosis using tests that are licensed in the United States. J Clin Microbiol.

[33] Katz DA, Golden MR, Stekler JD (2012). Use of a home-use test to diagnose HIV infection in a sex partner: a case report. BMC Res Notes.

[34] Bavinton BR, Brown G, Hurley M (2013). Which gay men would increase their frequency of HIV testing with home self-testing?. AIDS Behav.

